# The role of high-dose myeloablative chemotherapy with haematopoietic stem cell transplantation (HSCT) in children with central nervous system (CNS) tumours: protocol for a systematic review and meta-analysis

**DOI:** 10.1186/s13643-015-0155-7

**Published:** 2015-11-20

**Authors:** Caroline Main, Jayne S. Wilson, Simon P. Stevens, Aimee E. Houlton, Martin English, Pamela R. Kearns, Bob Phillips, Barry Pizer, Sophie Wilne, Keith Wheatley

**Affiliations:** Cancer Research UK Clinical Trials Unit (CRCTU), Institute of Cancer and Genomic Sciences, University of Birmingham, Birmingham, UK; Birmingham Children’s Hospital NHS Foundation Trust, Birmingham, UK; Centre for Reviews and Dissemination (CRD), University of York, York, UK; Alder Hey Children’s NHS Foundation Trust, Liverpool, UK; Queen’s Medical Centre, Nottingham University Hospitals’ NHS Trust, Nottingham, UK

**Keywords:** Children, Central nervous system tumours, High-dose chemotherapy, Haematopoietic stem cell transplantation, Systematic review

## Abstract

**Objectives:**

The objective of the study is to conduct a systematic review to compare the effects of high-dose chemotherapy (HDCT) with autologous haematopoietic stem cell transplantation (HSCT) versus standard-dose chemotherapy (SDCT) in children with malignant central nervous system (CNS) tumours.

**Methods:**

Standard systematic review methods aimed at minimising bias will be employed for study identification, selection and data extraction. Ten electronic databases will be searched, along with citation searching and reference checking. Studies assessing the effects of HDCT with HSCT in children with CNS tumours will be included. The outcomes are survival (overall, progression-free, event-free, disease-free), response rates, short- and long-term adverse events and health-related quality of life (HRQoL). Two reviewers will independently screen and select randomised and non-randomised controlled trials and controlled and uncontrolled observational studies for inclusion. Quality assessment will be tailored to the different study designs. Where possible data will be summarised using combined estimates of effect for the hazard ratio for survival outcomes and the risk ratio for response rates. A fixed effect model will be used; sub-group analyses and meta-regression will be used to explore potential sources of heterogeneity between studies.

**Discussion:**

Given the poor prognosis of malignant brain tumours in children in terms of survival and quality of life, this review will help guide clinical practice by summarising the current evidence on the use of high-dose myeloblative chemotherapy with stem cell support in children with CNS tumours.

## Background

Tumours of the central nervous system (CNS) represent a diverse group of neoplasms that account for approximately 25 % of all childhood cancers. They are the leading cause of cancer-related death in childhood and severe morbidity in survivors. High-grade gliomas (HGG) [glioblastoma multiforme (GBM) and anaplastic astrocytomas (AA)], diffuse intrinsic pontine gliomas (DIPG), primitive neuroectodermal tumours (PNETs, including medulloblastoma) and ependymoma constitute the majority of these malignant tumour types. To date, multimodal treatment involving surgery, radiotherapy and chemotherapy has formed the main stay of treatment for CNS tumours. However, the survival rate remains poor in some high-risk histological tumour types and for patients with residual, recurrent or disseminated disease. Therapeutic options in most of these patients are limited by previous chemotherapy and radiotherapy and the need to limit re-irradiation in second-line treatment due to the deleterious effects on the developing brain and spinal cord.

High-dose chemotherapy (HDCT) followed by haematopoietic stem cell transplantation (HSCT) has been used as frontline as well as salvage therapy in children with a variety of CNS malignancies [1–3]. This strategy is based on the principle of high-dose therapy facilitating better penetration of the blood-brain barrier (BBB) and an increase in the dose-response curve to chemotherapeutic compounds. Clinically, however, severe myelosuppression limits dose escalation unless the haematopoietic system can be rescued shortly after infusion. The use of HDCT followed by HSCT has proven to be feasible and a number of single arm phase II trials have assessed the effects of HDCT with HSCT in different groups of children with CNS tumours. These have included infants [4, 5] and children with newly diagnosed [6] or relapsed medulloblastoma [7, 8], HGG [9–12] and relapsed or progressive ependymoma [13]. The totality of the evidence related to using HDCT with HSCT in children with CNS tumours has however not been systematically assessed. This review therefore aims to assess the effects of HDCT with HSCT versus SDCT in children with malignant CNS tumours.

## Methods

Standard systematic review methodology aimed at minimising bias will be employed, and reporting will follow the Preferred Reporting Items for Systematic Reviews and Meta-Analyses (PRISMA) guidelines [14]. The protocol for this review is registered with PROSPERO (CRD42015020402). Available from http://www.crd.york.ac.uk/prospero/display_record.asp?ID=CRD42015020402).

### Data sources and searches

This review forms part of a wider work programme of systematic reviews which aim to assess the effects of different interventions for the treatment of CNS tumours in children, adolescents and young adults. Searches have therefore been conducted for studies examining the effects of surgery, radiotherapy, chemotherapy, immunotherapy, hormone therapy, biological therapies and imaging used alone or as part of a multi-modality treatment regimen for all types of paediatric brain tumours. No study design filters have been applied to the searches. Specific details of the searches conducted are detailed below.

Bibliographic databases: A comprehensive, broad search strategy was developed using a combination of medical subject headings (MeSH) and free text terms. The searches were limited by date from 1985 to November week 1, 2014. No language or publication status restrictions were applied, and ongoing studies were included.

The searches for published studies were undertaken using the following databases: MEDLINE (OvidSP); MEDLINE In-Process Citations and Daily Update (OvidSP); EMBASE (OvidSP); Cochrane Database of Systematic Reviews (CDSR) (Wiley); Cochrane Central Register of Controlled Trials (CENTRAL) (Wiley); CINAHL Plus (EBSCO); Database of reviews of effects [DARE (Centre for Reviews and Dissemination (CRD) website)] and Health Technology Assessment (HTA) (CRD website). The search strategy used for the MEDLINE search is reported in Appendix 1.

Grey literature, completed and on-going studies were identified by searches of NIH Clinical Trials (http://www.clinicaltrials.gov/); Current Controlled Trials (http://www.controlled-trials.com/) and WHO International Clinical Trials Registry Platform (ICTRP) (http://www.who.int/ictrp/en/).

Other sources: Experts in the field, from both the Project Advisory and Patient and Public Involvement (PPI) Groups were contacted with a list of identified studies to find out whether they had knowledge of any further studies that had not been retrieved by the electronic searches. Reference lists of all studies included in the present review will be checked and citation searching undertaken in order to identify any further studies not retrieved by the electronic searches.

All identified references were downloaded into Endnote X7 software for further initial assessment and handling. As a preliminary first stage to the broader set of reviews, inclusion screening on the basis of the population and broader set of applicable interventions was undertaken, with all included studies being ‘mapped’ by study design. Where flexibility is needed throughout the work programme for reference management and handling, Endnote software will be linked to bespoke Access databases in order facilitate sorting and manipulation of data items within indexed fields and abstracts.

### Inclusion/exclusion criteria

#### Population

Infants, children and young adults (up to age 25 years) with diagnoses of any type of malignant CNS tumour. These include but are not limited to high- and low-grade gliomas (HGG and LGG), diffuse intrinsic pontine glioma (DIPG), medulloblastoma, ependymoma, germ cell tumours, atypical teratoid rhabdoid tumour (AT/RT), primitive neuroectodermal tumours and pineoblastoma. Studies that include both children and adults within the relevant populations will be included provided results are reported separately for children (defined as up to the age of 25). Likewise, studies conducted in children with different tumour types (e.g. solid tumours) will also be included provided results are reported separately for CNS tumours.

#### Interventions

HDCT with HSCT. High-dose methotrexate will be excluded as the term high-dose is used in the context of needing supportive care with folinic acid rescue as opposed to conventional standard dose methotrexate.

Comparator (for controlled studies): Standard or dose intensive chemotherapy.

#### Outcomes

Overall survival (OS), progression-free survival (PFS), disease-free survival (DFS), event-free survival (EFS), response rates, short- and long-term adverse events and health-related quality of life (HRQoL). Response status data prior to and after HDCT regimens need to be reported where either multi-modal therapy regimens are being assessed or studies include populations with different types of tumours within the same study. Studies which do not report both baseline and post-HDCT response data will be excluded.

#### Study designs

Randomised controlled trials (RCTs), non-randomised comparative studies, single arm phase II trials, cohort studies, case-control studies and case series (both prospective and retrospective) will be included provided that ten or more participants have been included in studies that have assessed single tumour types and fifteen or more participants have been included in studies that have assessed ‘mixed’ CNS tumour types within the same study. Cross-sectional studies and multiple and single case reports will be excluded.

### Study selection

Study selection will be undertaken in two stages by two reviewers working independently. Titles and abstracts will be screened for inclusion/exclusion. Studies marked for inclusion by either reviewer will then undergo full independent text assessment. Any discrepancies will be resolved by recourse to the abstracts or full texts or through consensus with a third reviewer. A PRISMA flow chart illustrating the study selection process will be documented [14].

### Data extraction

Data will be recorded on a standard data extraction form and entered onto a bespoke computer database developed in either Access or Excel. The data will be extracted by one reviewer and checked by a second for accuracy. Any discrepancies will be resolved by recourse to the paper. Data from studies with multiple publications will be extracted and reported as a single study. Data will be extracted on general [study name, study group (if applicable), publication date(s), principal investigator/authors]; eligibility and study participants [e.g. tumour type and location; grade; age, prior treatment history]; intervention and comparator (if applicable); intervention(s) [drugs, doses, number of cycles, administration, concomitant therapy], methods of HSCT [including donor source and cell type(s)], study design (e.g. RCT, non-randomised comparative study, single-arm phase II trial, prospective or retrospective case series], length of follow-up and timing of outcome assessments; outcome measures (protocol specified—if available—and reported); side effects/toxicity, long-term adverse events and neurological outcomes; analysis methods [intention to treat (ITT) or per protocol] and author's conclusions. Outcomes for RCTs and observational studies will be recorded separately.

### Assessment of risk of bias in studies

The quality of RCTs will be assessed using the eight criteria in the Cochrane Collaboration’s tool for assessing risk of bias, which covers random sequence generation and allocation concealment (selection bias); blinding [(participants, personnel and outcome assessors), performance and verification bias]; completeness of outcome data (attrition bias); selective outcome reporting (reporting bias) and other sources of bias [15]. These criteria will be adapted for use with non-randomised comparative studies, with an assessment of baseline balance between groups being instigated instead of an assessment of the method of random sequence generation. For non-comparative studies, the six-point checklist developed by CRD, York for the assessment of observational studies will be utilised (https://www.york.ac.uk/crd/guidance/) [16]. This checklist asks whether the sample is representative of the usual clinical population to whom the results will be extrapolated, whether the inclusion criteria are explicit and individuals entered the study at similar points in their disease progression (selection bias), whether follow-up was long enough for important outcomes to occur and whether outcomes were assessed using objective criteria or assessed blinded (detection bias). If a sub-series has been undertaken, it also asks whether there was a sufficient description of the distribution of prognostic factors. As well as the risk of bias tool and CRD checklist, for all studies, there will be an assessment of the adequacy of the sample size, the use of concomitant treatments, treatment compliance, the use of objective outcomes and the timing of outcome assessments, the appropriateness of the statistical analysis and whether the author’s conclusions are justified and consistent with the results presented. External validity will also be assessed according to the ability of the reader to consider the applicability of the findings to a patient group in practice.

All assessment will be at the overall study level, not at the level of the individual outcomes. In addition to the methodological criteria listed above, the GRADE framework may be used to consider inconsistency between studies, precision of results, likelihood of publication bias and applicability of results to population(s) of interest [17]. Quality assessment will be undertaken by one reviewer independently and checked for accuracy by a second. Any disagreements will be resolved by recourse to the study paper(s), and a third reviewer will be consulted where necessary.

### Data synthesis and analysis

#### Narrative synthesis

A narrative synthesis of study results will be presented (including text, figures and tables), to provide adequate interpretation of study findings. Studies will be grouped by tumour type, study design, intervention, and treatment line (induction, consolidation, salvage). The outcomes considered include overall survival, PFS, DFS, EFS, response rates, adverse events and HRQoL. Therefore, outcomes will be expressed in terms of hazard ratios [HR; (adjusted or unadjusted)] and risk ratios (RR). All analyses will be conducted per outcome, including all studies that have reported data for the outcome.

#### RCTs

Where more than one RCT has addressed the same question and they are considered to be clinically similar (based on patient population and study treatments), results will be combined in a standard pair-wise meta-analysis using assumption free methods. All analyses will be carried out on an ITT basis where possible, using the HR or RR as appropriate. Heterogeneity of treatment effect, if present, will be investigated using the chi-squared test for heterogeneity and the *I*^2^ statistic [18]. Further sub-group analyses, to explore differences between the trials in terms of patient baseline characteristics such as tumour grade, prior treatments (dose, number of cycles) will be undertaken as necessary to investigate whether the treatment effect differs between patient sub-groups.

If feasible and significant heterogeneity is identified, this will be investigated using meta-regression. The following pre-specified factors will be investigated: quality of the primary studies; tumour type; prior treatment regimens (RT versus no RT); induction/consolidation/salvage therapy; dose and number of treatment cycles. All analyses will be conducted using RevMan (version 5.1) and STATA (STATA™ for Windows, version 10.1, Stata Corp; College Station, TX).

#### Assessment of small study effects

For each meta-analysis containing ten or more studies, the likelihood of small study effects and publication bias, namely the tendency for smaller studies to provide more positive findings, will be investigated though the construction of funnel plots and statistical tests for small study effects (such as the Peters Test) [19].

#### Non-randomised comparisons

Differences in outcome between studies will be assessed by formal statistical (e.g. chi-squared tests to compare response rates) and if the data permit, appropriate tests for time-to-event and continuous parameters. Graphical displays will be constructed in order to allow informal comparison of the results between studies. Differences in baselines characteristics of the patients that might explain any apparent differences in outcome will be examined.

Where both RCTs and non-randomised studies have assessed similar interventions within the same tumour type, a comparison of the effect sizes between the two different study designs will be conducted to evaluate any differential treatment effects by study design.

## Discussion

Although this methodology has been designed to be comprehensive and to minimise bias, we anticipate some limitations with this review. The evidence base is likely to be highly heterogeneous with different HDCT interventions evaluated as part of a multi-modality treatment regimen. Furthermore, these will be assessed in different patient populations as induction, consolidation or salvage therapy. Additionally, the majority of the evidence will come from single-arm phase II trials and small case series both of which have a strong risk of bias. It is therefore likely that any conclusions that can be drawn from the review will be tentative with a lot of uncertainty. However, given the paucity of the available evidence in order to inform treatment decision making in terms of the risk-benefit profile of different types of HDCT in the treatment of children with CNS tumours, it is very important to undertake this review.

To ensure that our findings have clinical impact on patients, their parents and the physicians who care for them, results will be disseminated broadly by presenting at scientific conferences, publishing in peer-reviewed journals, and through our established Patient and Public Involvement (PPI) partners who work for established high profile UK Brain Tumour Charities and our Clinical Steering Group. For completeness, a list of the individual’s and their affiliations involved in both of these groups is provided in Appendix 2.

## Appendix 1: Clinical Effectiveness Search Strategy

Medline (OvidSP): 1985—October Week 4 2014

1. Glioma/ or Brain Neoplasms/ or Meningioma/ or Glioblastoma/ or Astrocytoma/

2. ((brain or brainstem or intracranial or posterior fossa) adj3 (cancer* or carcinom* or tumour* or tumour* or neoplasm*)).mp.

3. (Astrocytoma* or Brain Stem Glioma* or Medulloblastoma*or Primitive Neuroectodermal Tumo?r* or ganglioneuroblastoma* or CNS neuroblastoma* or Ependymoblastoma or Medulloepithelioma or Pineal Parenchymal Tumour* or (Atypical Teratoid adj1 tumo?r*) or Oligoastrocytoma or ((Pilocytic or Gemistocytic) adj1 astrocytoma*) or ependymoma or primitive neuroectal tumo?r*).mp.

4. (((Diffuse fibrillary or Gemistocytic or Pilocytic Pilomyxoid Protoplasmic Subependymal giant cell) adj1 astrocytoma*) or Oligoastrocytoma or Oligodendroglioma or Oligoastrocytoma or Pleomorphic xanthoastrocytoma or ((astrocytoma or oligoastrocytoma or oligodendroglioma) adj1 astrocytoma*) or Glioblastoma or Gliomatosis cerebri or Gliosarcoma or ((diffuse intrinsic pontine glioma or low grade brain stem) adj1 glioma) or ((classic or desmoplastic or nodular or large cell or nodularity) adj1 medulloblastoma*) or Primitive Neuroectodermal Tumo?r* or ((ganglioneuroblastoma or neuroblastoma) adj 1central nervous system*) or Ependymoblastoma or Pineoblastoma or pineal parenchymal tumo?r* or (central nervous system adj1 atypical teratoid) or (central nervous system adj 1 rhabdoid tumo?r*) or Germinomas or ((immature or mature or malignant transformation) adj2 teratomas)).mp.

5. 1 or 2 or 3 or 4

6. exp Surgical Procedures, Operative/

7. surg*.mp.

8. debulk*.mp.

9. cytoreduc*.mp.

10. 6 or 7 or 8 or 9

11. (chemotherap* or antineoplastic agents or cytotoxic or alkylating agents or nitrosoureas or antimetabolite* or antitumor?r or ((antibod* or monoclonal) adj 3 Human*) or plant alkyloid* or (hormone* adj 1 agent*) or anthracycline* * or systemic therap*)).mp.

12. (Everolimus or Afinitor or Cetuximab or Erbitux or Bevacizumab or Avastin or Cediranib or Recentin or lomustine or CCNU or CeeNU or carmustine or BiCNU or Carustine or Ethylnitrosourea or Streptozocin or Sorafenib or Nexavar or tipifarnib or Zarnestra or Erlotinib or Tarceva or Sorafenib or Nexavar or temsirolimus or Torisel or Sunitinib or Sutent or irinotecan or Camptosar or Campto or Vandetanib or Caprelsa or Cabozantinib or Cometriq or XL184 or Axitinib or AG013736 or Inlyta).mp.

13. 11 or 12

14. exp Immunotherapy/ae, cl, ct, mt, mo, nu, px, st

15. exp Genetic Therapy/ae, cl, ct, mt, mo, nu, ut

16. exp Imaging, Three-Dimensional/ or exp Whole Body Imaging/ or exp Magnetic Resonance Imaging/

17. exp Tomography, Emission-Computed/ or exp Four-Dimensional Computed Tomography/ or exp Tomography/ or exp Tomography, Emission-Computed, Single-Photon/ or exp Positron-Emission Tomography/

18. 16 or 17

19. (radiation therapy or radiotherap* or intensity modulat* radiotherapy*or radiosurgery or radiation oncology or reduced boost volume radiotherap* or hyper fractionat* stereotactic radiotherap*or adjuvant radiotherap* or body radiotherap* stereotactic*or computer assisted radiotherap*or computer assisted radiotherap*planning or conformal radiotherap* or dosage* radiotherap* or dose fractionation* radiotherap* or high energy radiotherap*or implant radiotherap*or intensity or modulated radiotherap*or interstitial radiotherap*orimage guided radiotherap*or stereotactic*guid* radiotherap* or local therap*).mp.

20. 10 or 13 or 14 or 15 or 18 or 19

21. 5 and 20

22. (Response or overall survival or progression* free survival or event* free survival or time to recurrence or time to progression or disease* free interval* or endocrinopath* or ((growth or thyroid) adj 1 hormone adj 3 deficienc*) or ((glucocorticoid or gonadotropin) adj 3 deficienc*) or endocrine dysfuct* or (cardiac function* adj 3 impair*) or ataxia or spastic paresis or visual dysfunction or epilepsy or hemiparesis or neurolog* deficit*).mp.

23. 21 and 22

24. limit 23 to (year = ‘1985-Current’ and (‘newborn infant (birth to 1 month)’ or ‘infant (1 to 23 months)’ or ‘preschool child (2 to 5 years)’ or ‘child (6 to 12 years)’ or ‘adolescent (13 to 18 years)’ or ‘young adult (19 to 24 years)’) and humans)

## Appendix 2

**Table 1:** List of members of the Clinical Steering Group

Name	Speciality	Affiliation
Grant Holding Group		
Dr. Martin English	Consultant paediatric oncologist	Birmingham Children’s Hospital, Birmingham
Professor Pamela Kearns	Consultant paediatric oncologist	Birmingham Children’s Hospital, Birmingham
Dr. Bob Phillips	Consultant paediatric oncologist	Leeds Children’s Hospital, Leeds
Professor Barry Pizer	Consultant paediatric oncologist	Alder Hey Children's Hospital, Liverpool
Dr. Sophie Wilne	Consultant paediatric oncologist	Nottingham University Hospital, Nottingham
Clinical Steering Group Members		
Dr. Darren Hargrave	Consultant paediatric oncologist	Great Ormond Street Hospital, London
Professor David Walker	Consultant paediatric oncologist	School of Human Development, University of Nottingham
Dr. Heidi Traunecker	Consultant paediatric oncologist	University Hospital of Wales, Cardiff
Dr. Nicky Thorp	Consultant oncologist	Clatterbridge Centre for Oncology, Flintshire
Professor Richard Grundy	Paediatric neuro-oncology and cancer biology	School of Medicine, University of Nottingham
Professor Roger Taylor	Professor of clinical oncology	College of Medicine, Swansea University
Professor Simon Bailey	Consultant paediatric oncologist	Great North Children’s Hospital, Newcastle
Dr. Conor Mallucci	Consultant paediatric neurosurgeon	Alder Hey Children’s Hospital, Liverpool
Dr. Susan Picton	Consultant paediatric oncologist	Leeds Children’s Hospital, Leeds
Dr. Alison Evans	Head of research and policy	The Brain Tumour Charity
Rosemary Wormington	Head of support	Brain Tumour Support
Sharon Sambrook	Support co-ordinator	Brain Tumour Support

## Abbreviations

AA: anaplastic astrocytomas—a rare grade III tumour that develops from the neuroepithelial tissue
AT/RT: atypical teratoid rhabdoid tumour—an embryonal tumour that can occur anywhere in the central nervous system
BBB: blood-brain barrier—a highly selective permeable barrier that separates the circulating blood from the brain extracellular fluid in the central nervous system
CNS: central nervous system—the part of the nervous system consisting of the brain and the spinal cord
DIPG: diffuse intrinsic pontine gliomas—heterogeneous group of tumours occurring in the brainstem and cervicomedullary junction that are typically anaplastic astrocytomas or glioblastoma multiforme
GBM: glioblastoma multiforme—also known as glioblastoma or grade IV astrocytoma it it involves the glial cells and develops from the neuroepithelial tissues
HDCT: high-dose chemotherapy—an intensive drug treatment to kill cancer cells, but that also destroys the bone marrow and can cause other severe side effects. High-dose chemotherapy is usually followed by bone marrow or stem cell transplantation to rebuild the bone marrow
HGG: high-grade glioma—high-grade gliomas encompass the WHO grade III gliomas (anaplastic astrocytoma) and grade IV gliomas (glioblastome multiforme)
HSCT: haematopoietic stem cell transplantation—the intravenous infusion of haematopoietic stem cells usually derived from bone marrow, peripheral blood or umbilical cord blood to re-establish haematopoietic function in patients whose bone marrow or immune system is damaged or defective
PNETs: primitive neuroectodermal tumours—a malignant neural crest tumour
SDCT: standard dose chemotherapy—chemotherapy delivered within standard dosing schedules without the use of haematopoietic stem cell transplantation
